# Effects of ACEI/ARB or CCB use on atrial fibrillation in hypertensive patients following permanent pacemaker implantation

**DOI:** 10.3389/fcvm.2023.1191539

**Published:** 2023-06-22

**Authors:** Zhijie Liu, Ning Bian, Shaorong Wu, Yantao Cao, Yiting Su, Wenying Li, Hairui Li, Xianwu Lan, Can Jiang, Yiming Fan, Jun Guo, Dongdong Chen

**Affiliations:** ^1^ Department of Cardiology, The First Affiliated Hospital of Jinan University, Guangzhou, China; ^2^ Department of Nephrology, Shenzhen Hospital of Southern Medical University, Shenzhen, China

**Keywords:** atrial fibrillation, pacemaker implantation, hypertension, angiotensin-converting enzyme inhibitors (ACEI), angiotensin receptor blockers (ARB), calcium channel blockers (CCB)

## Abstract

**Aims:**

Permanent pacemaker implantation (PPI) combined with hypertension leads to a higher risk of new-onset atrial fibrillation (NOAF) for patients. Hence, it is essential to study how to reduce this risk. Currently, the effects of the two common anti-hypertensive drugs, angiotensin-converting enzyme inhibitors (ACEI)/angiotensin receptor blockers (ARB) and calcium channel blockers (CCB), on the risk of NOAF for such patients remain unknown. This study aimed to investigate this association.

**Methods:**

This single-center retrospective study included hypertensive patients with PPI and without prior history of AF/atrial flutter, heart valve disease, hyperthyroidism, etc. Patients were classified into ACEI/ARB group and CCB group based on their exposure drug information. The primary outcome was NOAF events that occurred within 12 months after PPI. The secondary efficacy assessments were the changes from baseline to follow-up in blood pressure and transthoracic echocardiography (TTE) parameters. A multivariate logistic regression model was used to verify our aim.

**Results:**

A total of 69 patients were finally included (51 on ACEI/ARB and 18 on CCB). Both univariate analysis [odds ratio (OR) 0.241, 95% confidence interval (CI) 0.078–0.745] and multivariate analysis (OR: 0.246, 95% CI: 0.077–0.792) demonstrated that ACEI/ARB were associated with a lower risk of NOAF compared to CCB. The mean reduction in left atrial diameter (LAD) from baseline was greater in ACEI/ARB group than in CCB group (*P *= 0.034). There was no statistical difference between groups in blood pressure and other TTE parameters after treatment.

**Conclusion:**

For patients with PPI combined with hypertension, ACEI/ARB may be superior to CCB in selecting anti-hypertensive drugs, as ACEI/ARB further reduces the risk of NOAF. One reason for this may be that ACEI/ARB improves left atrial remodelling such as LAD better.

## Introduction

Permanent pacemaker implantation (PPI), the most effective treatment for irreversible sinus node dysfunction or high-grade heart block, can reduce clinical symptoms, improve quality of life, and even increase survival rate of patients. However, PPI may also pose some potential risks to patients (1, 2). For example, the implanted pacemaker alters cardiac electrophysiology and hemodynamics, possibly leading to an increased risk of atrial fibrillation (AF) (2). As one of the most common cardiac arrhythmias, AF is associated with a higher risk of death, stroke, thromboembolism, and bleeding, even if the patients are given prompt treatment (3). There were studies showing that the incidence and prevalence of AF have increased steadily in recent years. Yet, postdiagnosis survival of patients has not improved (4, 5). Therefore, primary prevention to reduce the occurrence of AF is particularly important.

In addition to the impact of the pacemaker itself on the heart, some clinical comorbidities may further increase the risk of AF for patients. To identify the risk factors of new-onset AF (NOAF) after pacemaker implantation in elderly patients, Chen XL et al. conducted a retrospective study. The results showed that hypertension is the most significant risk factor for NOAF after pacemaker implantation (hazard ratio = 3.040, *P *= 0.00) (2). Hence, how to reduce the risk of NOAF in patients with pacemaker implantation combined with hypertension deserves study in depth.

A core treatment of hypertension is the use of anti-hypertensive drugs such as angiotensin-converting enzyme inhibitors (ACEI), angiotensin receptor blockers (ARB), and calcium channel blockers (CCB). Among these, ACEI/ARB have shown anti-arrhythmic effects that are useful as a part of the upstream therapy in managing AF both for primary and secondary prevention (6). This has been demonstrated in previous studies. For example, ACEI/ARB decreased the incidence of AF in patients with left ventricular dysfunction (7), in patients with end-stage renal disease undergoing dialysis (8), and in patients undergoing coronary artery bypass and/or valve surgery (9). Similarly, ACEI/ARB have been shown to reduce the risk of NOAF in patients with hypertension or pacemaker implantation (10, 11). However, in a study assessing whether ACEI/ARB could reduce the risk of postoperative AF in patients undergoing pacemaker implantation, the difference between the ACEI/ARB group and the control group was not statistically significant (12). It is worth noting that only 30% of the patients in this study had combined hypertension. CCB are another commonly prescribed anti-hypertensive drug class and have also been suggested to have positive effects on atrial electrical remodeling and AF (13).

Inconsistent findings exist in previous studies comparing the effects of the above two different anti-hypertensive drugs on AF. A cohort study of hypertensive patients showed a lower incidence of NOAF in the ACEI group compared to the CCB group (13). A meta-analysis also found that ARB was significantly superior to CCB in preventing AF (14). Nevertheless, another randomized trial indicated that in patients with paroxysmal AF and hypertension, ARB did not have an advantage over CCB in reducing the frequency of AF episodes (15). At present, the effects of ACEI/ARB or CCB on the risk of NOAF in patients with PPI and hypertension remain uncertain. Hence, this study aimed to investigate the association between these drugs and the risk of NOAF in such patients.

## Methods

### Study design and population

This was a single-center, retrospective cohort study retrieving patients' data from the electronic medical record of The First Affiliated Hospital of Jinan University between January 2012 and January 2018. Patients who had undergone PPI and combined with hypertension were eligible for inclusion if their drug prescription records included either ACEI/ARB or CCB. Concomitant β-blockers use or other types of anti-hypertensive drug use were allowed. To minimise the interference of confounding factors for this study, we excluded individuals fulfilling the following criteria: (i) prior history of AF or atrial flutter; (ii) any of the following heart diseases, including acute coronary syndrome, heart valve disease, cardiomyopathy, congenital heart disease, rheumatic heart disease, pulmonary heart disease, cardiac surgery.; (iii) abnormal thyroid function (hyperthyroidism); (iv) incomplete clinical data. The Research Ethics Committee of The First Affiliated Hospital of Jinan University approved the study (KY-2023-020) and authorized a waiver of informed consent from the included patients due to the retrospective observational character of this study.

### Data collection

The information on main exposure drugs (ACEI/ARB or CCB) and other drugs (e.g., β-blocker) use could be abstracted from the patient's prescription records. Moreover, we collect the following covariates for each patient: age, gender, diabetes mellitus, coronary heart disease (CHD), blood pressure, and the transthoracic echocardiography (TTE) parameters including left ventricular ejection fraction (LVEF), left atrial diameter (LAD), left ventricular diameter (LVD), interventricular septal thickness (IVST).

### Outcomes

The primary outcome was NOAF events that occurred within 12 months after pacemaker implantation. The diagnosis of NOAF was confirmed by professional physicians according to the patients' intracavitary electrocardiogram (ECG) recorded by the pacemaker and standard or ambulatory ECG. In addition, we predefined secondary efficacy assessment of changes from baseline to follow-up in blood pressure and TTE parameters to compare the therapeutic effects of ACEI/ARB and CCB on blood pressure and cardiac structure. In the subgroup analysis, we subdivided the ACEI/ARB patients into the ACEI and ARB groups and compared their NOAF events. Finally, the single ACEI and ARB groups were compared with the CCB group on the risk of NOAF, respectively.

### Statistical analysis

Statistical descriptions of patients' baseline characteristics and univariate analysis of primary outcome were first performed. Continuous variables that were normally or approximately normally distributed were presented as mean ± standard deviation (SD) and compared using independent-samples Student's *t*-test, or otherwise as median [interquartile range (IQR)] and compared using the Mann–Whitney *U*-test. Categorical variables were presented as frequencies and percentages and compared with *χ*^2^ or Fisher's exact test. Variables with *P *< 0.2 in the univariate analysis were included in a multivariate logistic regression analysis. Besides, we performed an additional analysis adjusting for all covariates to eliminate their potential impacts on the results maximally. In the secondary efficacy assessment, independent-samples Student's *t*-test or Mann–Whitney *U*-test was used for comparison of the changes in blood pressure and TTE parameters after treatment between the groups. Comparisons of the primary outcome between the groups in the subgroup analysis were performed using the *χ*^2^ or Fisher's exact test. The normality of data was assessed for all continuous variables using Shapiro–Wilk test and graphical test (i.e., histograms and Q-Q plots). The odds ratio (OR) and their 95% confidence intervals (CI) were used as effect values to assess the correlation between study variables and outcomes. We considered a *P*-value of <0.05 statistically significant. All statistical processes were completed by SPSS 27 software.

## Results

**Figure 1:**
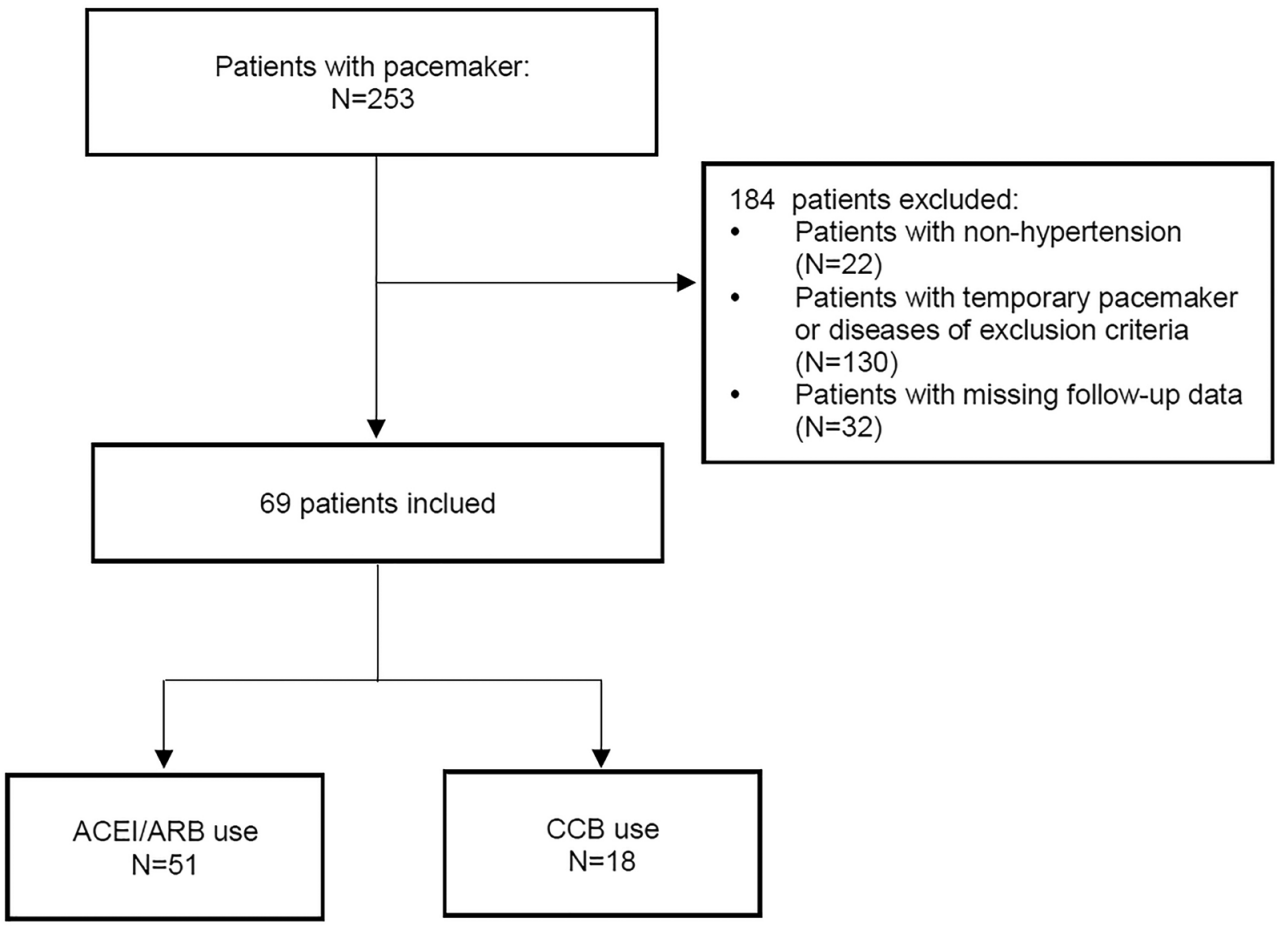
The patients’ flow chart of this study. ACEI, angiotensin-converting enzyme inhibitor; ARB, angiotensin-receptor blocker; CCB, calcium channel blocker.

Among a total of 253 patients receiving pacemaker implantation, 69 were finally included according to the inclusion and exclusion criteria (51 on ACEI/ARB and 18 on CCB). The patients' flow chart is explicitly shown in Figure 1. All patients were treated with PPI and dual-chamber pacing for sick sinus syndrome (SSS) or third-degree atrioventricular block (AVB). The ventricular pacemaker electrodes were all placed in the mid-low position of the right ventricular septum. The baseline characteristics of the patients in both groups are depicted in Table 1. Overall, there were no statistically significant differences between the two groups in terms of gender, age, history of diabetes mellitus, history of CHD, systolic blood pressure (SBP), diastolic blood pressure (DBP), LVEF, LAD, LVD, IVST, and β-blocker use (*P *> 0.05).

**Table 1:** Baseline clinical characteristics of study population.

Characteristics	Anti-hypertensive drug use		*P*-value
	ACEI/ARB	CCB	
Age (Years)	72.47 ± 9.67	68.44 ± 10.35	0.141
Male (*n* %)	19 (37.25%)	9 (50.00%)	0.344
Diabetes (*n* %)	20 (39.22%)	6 (33.33%)	0.658
SBP (mmHg)	175.43 ± 17.40	169.61 ± 13.64	0.203
DBP (mmHg)	86.53 ± 11.02	87.83 ± 10.17	0.661
CHD (*n* %)	19 (37.25%)	7 (38.89%)	0.902
LVEF (%)	62.14 ± 7.60	63.22 ± 5.77	0.583
LAD (mm)	38.92 ± 5.83	37.00 ± 4.94	0.217
LVD (mm)	44.73 ± 4.60	44.00 ± 4.37	0.562
IVST (mm)	10.94 ± 1.80	10.83 ± 1.20	0.815
β-blocker use	30 (58.82%)	10 (55.56%)	0.809

ACEI, angiotensin-converting enzyme inhibitor; ARB, angiotensin-receptor blocker; CCB, calcium channel blocker; SBP, systolic blood pressure; DBP, diastolic blood pressure; CHD, coronary heart disease; LVEF, left ventricular ejection fraction; LAD, left atrial diameter; LVD, left ventricular diameter; IVST, interventricular septal thickness.

### Primary outcome: AF after pacemaker implantation

**Figure 2:**
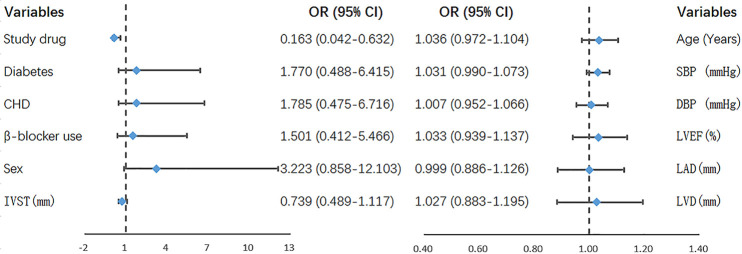
Parameters in the multivariate logistics regression model that used all the variables. SBP, systolic blood pressure; DBP, diastolic blood pressure; CHD, coronary heart disease; LVEF, left ventricular ejection fraction; LAD, left atrial diameter; LVD, left ventricular diameter; IVST, interventricular septal thickness; NOAF, new-onset atrial fibrillation; OR, odds ratio; 95% CI, 95% confidence interval.

The results of univariate and multivariate analyses of the primary outcome are shown in Table 2. The variables with *P *< 0.2 in univariate analysis were included in the multivariate logistic regression model, including study drug group (ACEI/ARB group or CCB group), history of CHD, and gender. The results showed that the study drug was the only variable influencing the risk of NOAF after PPI (OR: 0.246, 95% CI: 0.077–0.792, *P *= 0.019). ACEI/ARB were associated with a significant risk reduction in NOAF compared to CCB. This difference remained statistically significant after adjustment for all clinical covariates from Table 1 (OR: 0.163, 95% CI: 0.042–0.632, Figure 2).

**Table 2:** Univariate and multivariate analysis of primary outcome.

Categorical variable	Incidence of NOAF, % (no. of patients/total no. of patients)		Univariate analysis		Multivariate analysis	
			OR (95% CI)	*P*-value	OR (95% CI)	*P*-value
Study drug	ACEI/ARB	CCB	0.241 (0.078–0.745)	0.011	0.246 (0.077–0.792)	0.019
	27.5% (14/51)	61.1% (11/18)				
Diabetes	Yes	No	1.167 (0.425–3.199)	0.764	-	-
	38.5% (10/26)	34.9% (15/43)				
CHD	Yes	No	1.978 (0.721–5.425)	0.182	2.302 (0.769–6.886)	0.136
	46.2% (12/26)	30.2% (13/43)				
β-blocker use	Yes	No	1.140 (0.420–3.093)	0.797	–	–
	37.5% (15/40)	34.5% (10/29)				
Sex	Male	Female	2.094 (0.769–5.705)	0.145	2.143 (0.726–6.325)	0.168
	46.4% (13/28)	29.3% (12/41)				
Continuous variable	Mean ± SD value		Univariate analysis			
	NOAF	No NOAF	*P*-value			
Age (Years)	71.64 ± 8.18	71.30 ± 10.90	0.891		–	–
SBP (mmHg)	175.48 ± 15.12	173.02 ± 17.50	0.558		-	-
DBP (mmHg)	86.68 ± 9.34	86.98 ± 11.57	0.913		–	–
LVEF (%)	63.72 ± 6.02	61.68 ± 7.68	0.258		–	–
LAD (mm)	38.52 ± 4.73	38.36 ± 6.15	0.913		–	–
LVD (mm)	44.84 ± 4.73	44.36 ± 4.44	0.677		–	–
IVST (mm)	10.64 ± 1.55	11.07 ± 1.72	0.307		–	–

ACEI, angiotensin-converting enzyme inhibitor; ARB, angiotensin-receptor blocker; CCB, calcium channel blocker; SBP, systolic blood pressure; DBP, diastolic blood pressure; CHD, coronary heart disease; LVEF, left ventricular ejection fraction; LAD, left atrial diameter; LVD, left ventricular diameter; IVST, interventricular septal thickness; NOAF, new-onset atrial fibrillation; OR, odds ratio; 95% CI, 95% confidence interval.

### Predefined secondary efficacy assessment

**Figure 3:**
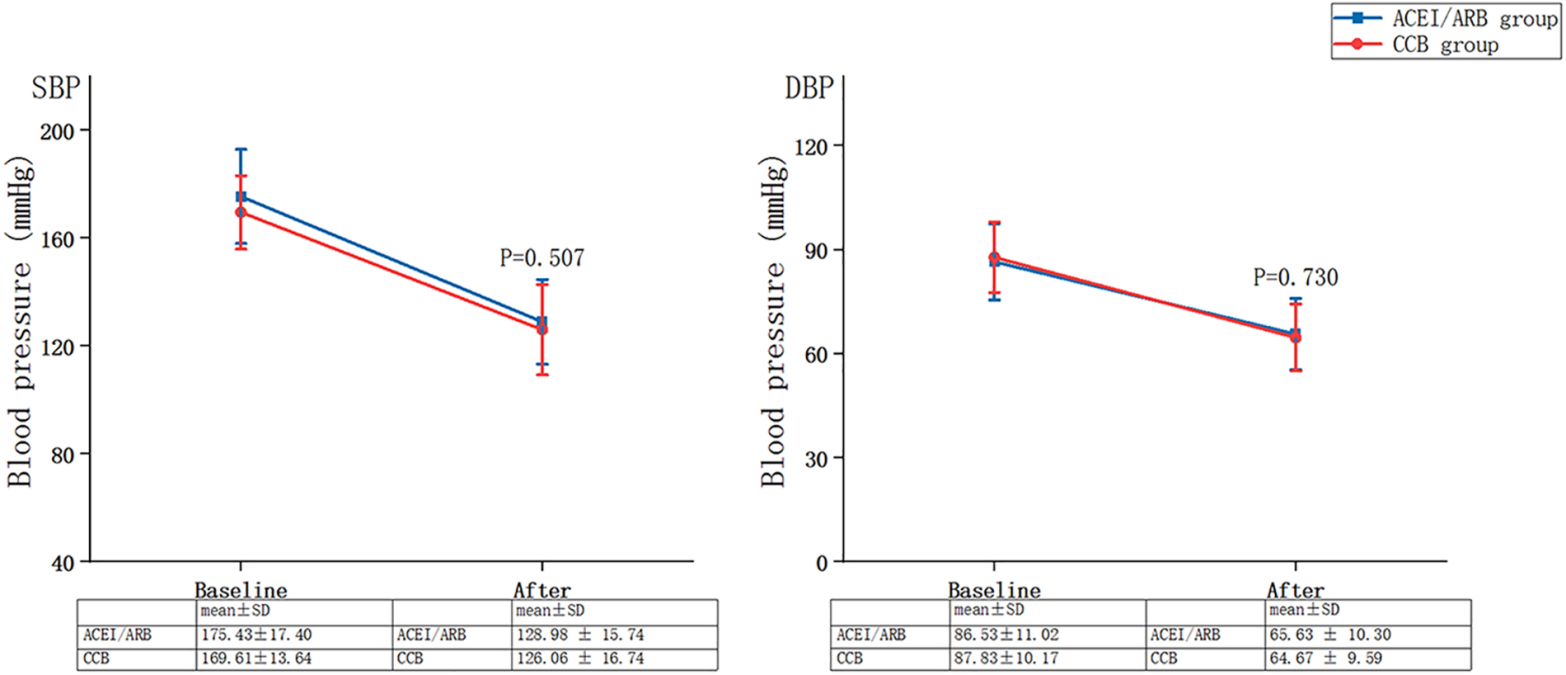
Changes in blood pressure between baseline and follow-up for the ACEI/ARB and CCB groups. SBP, systolic blood pressure; DBP, diastolic blood pressure; SD, standard deviation; ACEI, angiotensin-converting enzyme inhibitor; ARB, angiotensin-receptor blocker; CCB, calcium channel blocker.

Figure 3 summarises the changes in blood pressure between baseline and follow-up for the ACEI/ARB and CCB groups: SBP of no statistical difference between the two groups after treatment (ACEI/ARB group: from 175.43 ± 17.40 mmHg to 128.98 ± 15.74 mmHg vs. CCB group: from 169.61 ± 13.64 mmHg to 126.06 ± 16.74 mmHg, *P *= 0.507), and DBP of no statistical difference between the two groups after treatment (ACEI/ARB group: from 86.53 ± 11.02 mmHg to 65.63 ± 10.30 mmHg vs. CCB group: from 87.83 ± 10.17 mmHg to 64.67 ± 9.59 mmHg, *P *= 0.730).

**Figure 4:**
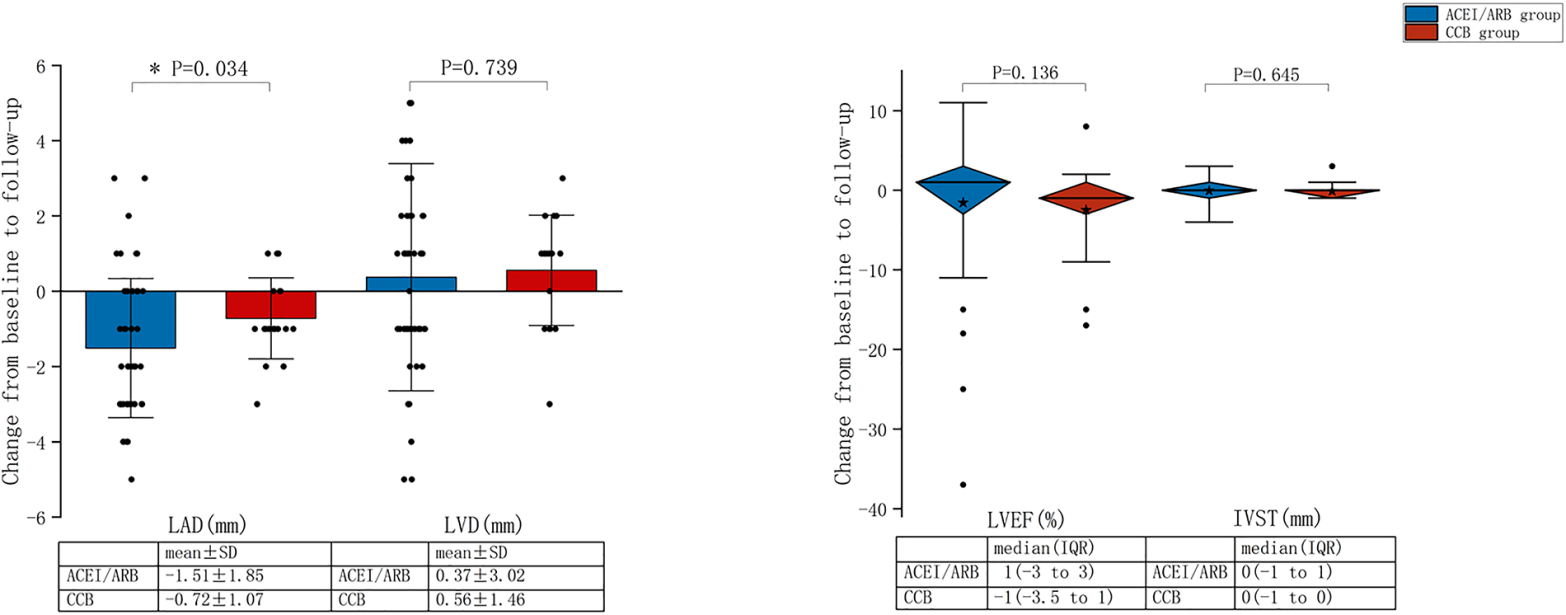
Changes from baseline to follow-up in the transthoracic echocardiography parameters for the ACEI/ARB and CCB groups. LVEF, left ventricular ejection fraction; LAD, left atrial diameter; LVD, left ventricular diameter; IVST, interventricular septal thickness; SD, standard deviation; IQR, interquartile range; ACEI, angiotensin-converting enzyme inhibitor; ARB, angiotensin-receptor blocker; CCB, calcium channel blocker.

Figure 4 summarises the changes in parameters of TTE from baseline to follow-up for the ACEI/ARB and CCB groups. The mean reduction in LAD before and after treatment was 1.51 ± 1.85 mm in the ACEI/ARB group and 0.72 ± 1.07 mm in the CCB group, with a statistically significant difference between the two groups (*P *= 0.034). There was no statistical difference in LVD, LVEF, and IVST (*P *> 0.05).

### Subgroup analyses

**Figure 5:**
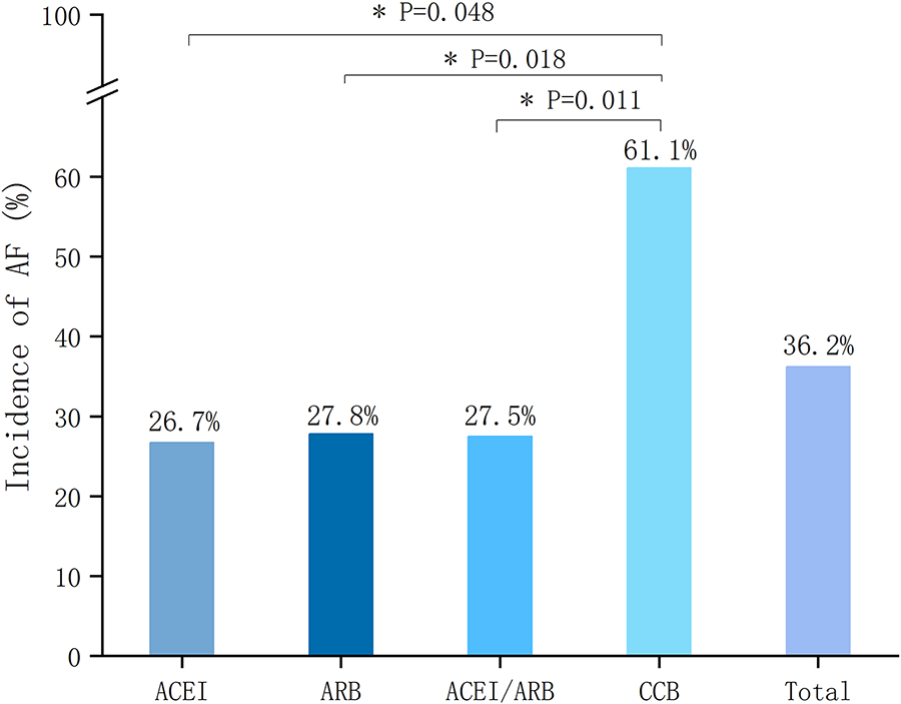
Incidence of NOAF according to different treatment groups. NOAF, new-onset atrial fibrillation; ACEI, angiotensin-converting enzyme inhibitor; ARB, angiotensin-receptor blocker; CCB, calcium channel blocker.

A total of 15 patients in the single ACEI group, of which 4 cases developed NOAF (26.7%). A total of 36 patients in the single ARB group, of which 10 cases developed NOAF (27.8%). There was no statistically significant difference in the incidence of NOAF between the above two groups (*P *> 0.05). The differences were both statistically significant between single ACEI or ARB group and CCB group (61.1%) (*P *= 0.048, *P *= 0.018, respectively). The comparison of the incidence of NOAF between groups is shown in Figure 5.

## Discussion

The main findings of this single-center retrospective cohort study are that (i) the effects of ACEI/ARB and CCB were different in reducing the risk of postoperative NOAF in patients with PPI and hypertension. ACEI/ARB was more effective in reducing this risk in such patients compared to CCB; (ii) a more pronounced reduction in LAD by ACEI/ARB suggests that ACEI/ARB produced more significant benefits in terms of reversing atrial remodelling than CCB.

The primary mechanism by which pacemaker implantation leads to a significant increase in the incidence of AF is generally considered directly related to the pathological remodelling of atrial structure and electrophysiology (16). In addition, inflammation is another important mechanism. Many previous studies have shown that inflammation is associated with AF closely. The exogenous stimulation from the pacemaker implantation induce an inflammatory response, which could lead to the increased release of inflammatory mediators such as interleukin-6 (IL-6), tumor necrosis factor-alpha, and C-reactive protein, thereby downregulating the expression of cellular junction proteins and promoting abnormity in cardiac electrical conductivity. The rest of mechanisms also include autonomic nervous disorder (like sympathetic activation), etc (17).

On the other hand, as an important independent risk factor contributing to the development of AF, hypertension could also lead to a greatly increased risk of AF by the following possible pathophysiological mechanisms (18, 19): (i) hemodynamic mechanisms: The left ventricle undergoes pathological changes such as thickening and stiffening of the ventricular wall to accommodate the increased afterload, which results in progressive impairment of left ventricular diastolic function. Pressure is then transmitted upwards to the left atrium, which is stretched by increasing pressure and volume, with subsequent left atrial remodelling including fibroblast proliferation, extracellular matrix alterations, myocyte hypertrophy, and the like. The muscle bundles of remodelled left atrial can become disordered on interconnections, leading to a shortened left atrial refractory period, unidirectional conduction block and reentry phenomena, and ultimately triggering AF; (ii) neurohumoral mechanisms: Under the condition of hypertension, both the renin-angiotensin-aldosterone system (RAAS) and the sympathetic nervous system tend to be over-activated. The former induces myocardial fibrosis by increasing the secretion of Angiotensin II and aldosterone, altering the expression of ion channels and thus increasing the risk of AF. The latter has also been shown to play an important role in the development of AF in preclinical studies in which sympathetic denervation improved electrophysiological parameters and reduced the percentage of AF relapse; (iii) the other possible mechanisms include atrial subtle electrophysiological alterations, obstructive sleep apnea (OSA), etc.

Although all studies included patients receiving PPI for SSS or AVB, an additional criterion combined with hypertension resulted in a significantly higher overall incidence of NOAF in our study (36.2%, follow-up one year, Figure 4) than in previous prospective (26%, mean follow-up 2.46 years) or retrospective trials (24.5%, mean follow-up 1.72 years) (2, 20). This is a finding that is consistent with previous study (2) (i.e., hypertension is a vital risk factor for NOAF after pacemaker implantation and further increases this risk). Moreover, the above-mentioned mechanisms of pacemaker implantation and hypertension leading to the development of AF could explain this finding.

The previous study has indicated four times increased risk of mortality for patients with AF compared with the general population (21). In recent years, some of those innovation milestones in AF treatment (e.g., advances in invasive techniques for radiofrequency ablation and the development of novel anticoagulant drugs) have had an impact on relieving patients' symptoms or preventing thrombotic strokes. However, the overall mortality rate of AF patients has not improved, and even, in fact, has risen (22). Given that, It is essential to study how to reduce the higher risk of NOAF in patients with PPI combined with hypertension. The key to the treatment of hypertension is the choice of anti-hypertensive drugs. As each patient's situation and problem are different, a critical principle in clinical medication selection is individualized treatment (23). Therefore, in addition to the effect of anti-hypertensive drugs on blood pressure, the appropriate hypertension treatment regimen for the patient should consider its efficacy on the other clinical comorbidity(ies) of patient. We have mentioned several relevant studies comparing the effects of ACEI/ARB and CCB on AF above. Nevertheless, to the authors' knowledge, there are no studies to assess the effect of ACEI/ARR or CCB on NOAF in patients with PPI combined with hypertension. In other words, whether ACEI/ARB or CCB use can reduce the risk of NOAF occurrence after PPI in hypertension patients remains unknown and should be researched.

Anti-hypertensive drugs can reduce the risk of AF by reversing hemodynamic mechanisms caused by hypertension, which seems to be the main mechanism explaining certain positive effects of CCB on AF (10, 14). By contrast, purely hemodynamic effect is insufficient to account for the AF risk reduction efficacy of ACEI/ARB, suggesting that it needs to be explained in combination with more effective targets (24). As classical RAAS inhibitors, ACEI/ARB have been shown to be effective in inhibiting atrial fibrosis, controlling inflammation (reducing, for instance, inflammatory mediators of IL-6 and C-reactive protein), modulating ion channels, and preventing cardiac electrical remodeling (25). Besides, it has been demonstrated that ACEI/ARB can also be beneficial on cardiovascular autonomic nerves and appear more pronounced in disease state with sympathetic over-activity (26). Through regulating the direction of metabolism, ACEI/ARB can increase Ang-(1-7) production as well (26). Ang-(1-7) might become a promising target for OSA-related hypertension considering its role of alleviating or reversing a series of pathological alterations caused by chronic intermittent hypoxia, the main pathophysiological mechanism of OSA and a contribution for the adverse cardiovascular consequences of OSA (27, 28).

Accordingly, ACEI/ARB may more comprehensively inhibit the underlying pathophysiological mechanisms by which hypertension leads to an increased risk of NOAF after PPI, and thus exert a better effect compared to CCB. This hypothesis was confirmed in our study. We observed a significant LAD reduction in the ACEI/ARB group compared to the CCB group in case of virtually identical blood pressure after treatment in both groups. At the same time, the risk of NOAF was remarkably lower in the ACEI/ARB group than in the CCB group, and this difference was still statistically significant after adjusting for relevant covariates. Based on these, we propose a possibility: ACEI/ARB may be superior to CCB in selecting anti-hypertensive drugs for patients with PPI and hypertension, as ACEI/ARB reduce the risk of NOAF further based on the same anti-hypertensive effect. One of the underlying reasons behind this may be that ACEI/ARB improves or reverses left atrial remodelling via more mechanisms. In a word, our study provides possible evidence for the clinical choice of medication for such individuals.

Outside of the foregoing main findings, another discovery inconsistent with previous studies in our subgroup analysis is also interesting. The therapeutic targets of ACEI and ARB are different theoretically, even though they are both RAAS inhibitors. As a result, their actual effects may vary a little, too. For example, direct inhibition of Angiotensin II production by ACEI simultaneously antagonizes AT1 receptor (causing myocardial fibrosis, promoting vasoconstriction, etc.) and AT2 receptor (attenuating myocardial fibrosis, mediating vasodilation, etc.). By comparison, the inhibition point of ARB is further downstream where ARB selectively antagonizes AT1 receptor, and then a feedback mechanism meanwhile increases AT2 activation (29, 30). A real-world study is consistent with this. Lin D et al. (29) found that ARB reduced the risk of AF after pacemaker implantation among elderly patients, while ACEI did not. For this reason, they suggested that ARBs might be more effective than ACEIs in preventing AF episodes after pacemaker implantation in elderly patients. Whereas our subgroup analysis showed that both ACEI and ARB were associated with a reduced risk of AF after pacemaker implantation in hypertensive patients, and there was no significant difference between them. Noteworthily, Lin D et al. and our conclusions were limited by the nature of single-center, small sample size, and retrospection. In consequence, future multicentre, larger prospective studies are needed to confirm whether ACEI and ARB differ in reducing the risk of AF after pacemaker implantation in different patients.

## Limitation

Our study has several limitations. First, this was a single-center study with a small sample size for inclusion, which may have partly influenced the statistical power needed to determine a significant difference between ACEI/ARB and CCB in the risk of NOAF for hypertensive patients after PPI. Second, although we excluded, to the greatest extent possible, confounding factors potentially affecting results, it is inevitable that a retrospective study introduces some bias. So there may still be some residual confounders in this study that cannot be fully excluded. Third, since the factors contributing to the occurrence of AF are sufficiently complex, omissions may still exist even though we have considered some of the most important. Fourth, the dose-response analysis in ACEI/ARB or CCB users was not conducted as the limited sample size.

## Conclusions

The findings of this retrospective study suggested that ACEI/ARB provided greater effects on reducing the risk of NOAF compared to CCB for patients with PPI combined with hypertension, which may be explained by that ACEI/ARB improves or reverses the left atrial remodelling such as LAD better. In addition, we found no significant difference between ACEI and ARB in their efficacy in reducing NOAF occurrence. However, more prospective, randomized controlled studies are needed to confirm our findings.

## Data Availability

The data analyzed in this study is subject to the following licenses/restrictions: Further inquiries can be directed to the corresponding authors. Requests to access these datasets should be directed to Dongdong Chen, 449244049@qq.com.

## Key points

- This retrospective cohort study in 69 patients with PPI combined with hypertension suggests that ACEI/ARB provided more excellent effects on reducing the risk of NOAF compared to CCB.
- ACEI/ARB produce more significant benefits in improving left atrial remodelling (such as a more pronounced reduction in LAD) than CCB.
- The subgroup analysis shows no significant difference between ACEI and ARB in terms of their efficacy in reducing NOAF occurrence after pacemaker implantation in hypertensive patients.
